# Mixed Methods Evaluation - A Chinese Cancer Screening Program

**DOI:** 10.1093/geroni/igaa057.2970

**Published:** 2020-12-16

**Authors:** Su-I Hou

**Affiliations:** University of Central Florida, Orlando, Florida, United States

## Abstract

This paper introduces the rapidly growing modern mixed methods research (MMR) and its application in a Chinese cancer screening program. While some previous researchers have incorporated quantitative and qualitative data in research, recent mixed methods developments have provided significant clarity that can guide those new to the MMR field. Understanding the context for using MMR and examining a complex mixed methods evaluation study in Taiwan can help illustrate opportunities for and application of mixed methods in Asians. The Taiwan Cervical Cancer Screening Education Program is used as an exemplar of a multi-phase complex mixed methods evaluation study showcasing various MMR designs. These include an exploratory sequential design to develop culturally sensitive study instrument, iterative concurrent and sequential mixed methods for intervention mapping, and an embedded mixed methods evaluation design to assess impact. Visual diagrams are introduced to facilitate communication of mixed methods design procedures and products in each phase.

